# Efficacy of a numerical value of a fixed-effect estimator in stochastic frontier analysis as an indicator of hospital production structure

**DOI:** 10.1186/1472-6963-12-334

**Published:** 2012-09-22

**Authors:** Hiroyuki Kawaguchi, Hideki Hashimoto, Shinya Matsuda

**Affiliations:** 1 Economics Faculty, Seijo University, 6-1-20 Seijo, Setagaya-ku, Tokyo, Japan; 2 Graduate School of Medicine, The University of Tokyo, 7-3-1 Hongo, Bunkyo-ku, Tokyo, Japan; 3 Department of Public Health, University of Occupational and Environmental Health, 1-1 Iseigaoka, Yahatanishi-ku, Kitakyushu, Fukuoka, Japan

**Keywords:** Casemix classification, Efficiency estimation, Fixed effects

## Abstract

**Background:**

The casemix-based payment system has been adopted in many countries, although it often needs complementary adjustment taking account of each hospital’s unique production structure such as teaching and research duties, and non-profit motives. It has been challenging to numerically evaluate the impact of such structural heterogeneity on production, separately of production inefficiency. The current study adopted stochastic frontier analysis and proposed a method to assess unique components of hospital production structures using a fixed-effect variable.

**Methods:**

There were two stages of analyses in this study. In the first stage, we estimated the efficiency score from the hospital production function using a true fixed-effect model (TFEM) in stochastic frontier analysis. The use of a TFEM allowed us to differentiate the unobserved heterogeneity of individual hospitals as hospital-specific fixed effects. In the second stage, we regressed the obtained fixed-effect variable for structural components of hospitals to test whether the variable was explicitly related to the characteristics and local disadvantages of the hospitals.

**Results:**

In the first analysis, the estimated efficiency score was approximately 0.6. The mean value of the fixed-effect estimator was 0.784, the standard deviation was 0.137, the range was between 0.437 and 1.212. The second-stage regression confirmed that the value of the fixed effect was significantly correlated with advanced technology and local conditions of the sample hospitals.

**Conclusion:**

The obtained fixed-effect estimator may reflect hospitals’ unique structures of production, considering production inefficiency. The values of fixed-effect estimators can be used as evaluation tools to improve fairness in the reimbursement system for various functions of hospitals based on casemix classification.

## Background

Increasing medical expenditure and threatened financial sustainability of healthcare systems are common policy issues in developed and developing countries, in the context of technological innovation and population aging. The healthcare system in Japan also faces a serious financial threat because of rapid population aging and long-lasting economic stagnation since the 1990s. Meanwhile, societal demand for quality healthcare is ever increasing. Consequently, improvements in the efficiency of healthcare provision are urgently needed to sustain the healthcare system in Japan.

One of the most popular methods of improving economic efficiency of service production in acute-care hospitals is the inclusive payment system based on casemix classification. Since the introduction of the system to the U.S. Medicare service in 1983, several countries including Japan have adopted varying types of inclusive payment schemes [1]. Inclusive payment forces hospital providers to efficiently allocate their resources and maximize their productivity under given resources by sharing the financial risk [2]. Too often, inclusive payment requires complementary adjustment to account for hospitals’ external conditions that are not amenable to efficiency management, such as teaching duties, research and development, and local disadvantages relating to the socio-economic environment. For example, U.S. Medicare adopts adjustment factors according to the number of residents per hospital bed, local price index, and local economic deprivation related to the availability of free care [3]. It is challenging, however, to discriminate such unique components of hospital production structures and their production inefficiency.

In this study, we employ a new method to estimate unique components of the hospital function structure, separately of estimations of production efficiency, using a new statistical model and panel datasets.

### Previous studies on the estimation of hospital production efficiency

A number of studies have estimated the technical efficiency of hospital production [4]. Among them, stochastic frontier analysis (SFA) was often preferred by econometricians because it can account for measurement error, and is robust against outlying observations [5]. Original SFA modeled a hospital’s efficiency independently of random error. However, the estimated product efficiency was still confounded by each hospital’s unique structural component of production function, related to location, status related to teaching, research, and technology advancement, which are likely to be time-invariant. Greene proposed the true fixed-effect model (TFEM) to explicitly account for such unobserved and invariant heterogeneity across hospitals [6,7]. The advantage of the TFEM is its ability to absorb unobserved heterogeneity without time variations by setting dummy variables for individual samples.

Several previous studies have adopted the TFEM to estimate hospital production efficiency. Jacobs, Smith, and Street (2006) were the first to adopt the TFEM in the field of health care to estimate hospital efficiency in the United Kingdom [8]. There were subsequent studies in Japan [9,10]. However, these studies simply used the number of outpatient, inpatient, or emergency cases as the production output, ignoring differences in the patient’s severity and treatment difficulties, which are likely to bias the estimation [11]. In addition, with the exception of Kawaguchi (2008), previous studies failed to adjust for the quality of care. Kawaguchi (2008) used the level of nurse staffing as a proxy of quality care, though still failed to use a direct measure of quality of care. In the current study, we overcome these limitations by taking advantage of a newly available casemix database that contains detailed information on the patient’s co-morbidity and disease severity, and relative resource utilization as used in Kawaguchi, Hashimoto and Matsuda (2010) [12].

Furthermore, estimated fixed effects unique to each hospital were not specifically analyzed in the previous studies. We expect that the value of a fixed-effect parameter should reflect the unobserved and time-invariant heterogeneity of each hospital’s unique structure of production. We hypothesized that the obtained fixed effect should have a significant association with hospital functions regarding teaching and technology advancement, and specific local conditions such as regional demographic characteristics.

## Methods

### Sample

In our analysis, we used a large casemix database based on Japanese diagnosis-related grouping called the Diagnosis Procedure Combination (DPC) [13]. The DPC was introduced to the Japanese social insurance system in 2003 for reimbursement of 82 special-function hospitals, consisting of main branches of university hospitals, and two national centers specializing in cancer and cardiovascular diseases [13]. The system has been extended to a wider spectrum of acute-care hospitals, and an additional 359 hospitals participated in 2006. Owing to limited data availability, we used a balanced panel dataset collected between 2005 and 2007 from 127 hospitals. The participating hospitals submitted anonymous data of discharged cases to a research group funded by the Ministry of Health, Labour and Welfare. The collection and use of data were approved by the Internal Review Board of the institute that the last author was affiliated with. The sample hospitals had an average of 600 beds, a relatively large number for Japanese hospitals.

### Analytical model of hospital technical efficiency

Economic efficiency can be assessed in terms of technical efficiency and allocative efficiency. In this study, we estimated the technical efficiency of Japanese hospitals according to their production function, or the extent of production for given resources. We chose not to assess allocative efficiency using price information, because Japanese hospitals are under rigid price control by the government, and operated at low cost [14]. Besides, hospital cost data are limited in Japan.

Following a conventional production function, Equations (1) and (2) are our analytical models, where *Y* is the production output, *L* is labor input, and *K* is capital input.

(1) $$\text{ln}Y_{\text{it}}=\text{ln}\alpha +\beta_{1}\text{ln}K_{\text{it}}+\beta_{2}\text{ln}L_{\text{it}}+v_{\text{it}}-u_{\text{it}}$$

(2) $$\begin{array}{l} \text{ln}Y_{\text{it}}=\alpha_{i}+\beta_{1}\text{ln}K_{\text{it}}+\beta_{2}\text{ln}L_{\text{it}}+\beta_{3}\left(\text{ln}L_{\text{it}}\right)\left(\text{ln}K_{\text{it}}\right)\\ \;+\frac{1}{2}\beta_{4}{\left(\text{ln}K_{\text{it}}\right)}^{2}+\frac{1}{2}\beta_{5}{\left(\text{ln}L_{\text{it}}\right)}^{2}+v_{\text{it}}-u_{\text{it}} \end{array}$$

$$v_{\text{it}}\sim N\left[0,\sigma_{v}^{2}\right],\;u_{\text{it}}=|U_{\text{it}}|,\;U_{\text{it}}=\left[0,\sigma_{u}^{2}\right]$$

$v_{\text{it}}$ is the error term, u_it_ is the efficiency score, and α_*i*_ is a time-invariant hospital-specific fixed effect.

Both models assume that efficiency is time-variant. Equation (1) follows the conventional Cobb–Douglas function, while Equation (2) is a more flexible model having a translog function. We adopted JLMS formulation to compute the efficiency measure Jondrow et al. (1982) [[15]].

### Variables for the efficiency estimation model

We treated the number of physicians as labor input (L) because physicians are responsible for treatment outcomes, and almost all Japanese hospitals adopt a closed system where physicians are employed in-house [14]. The number of full-time-equivalent physicians at each hospital was obtained from various published sources [16]. In Japanese law for the provision of health care, the placement and number of health care professionals, including doctors and nurses, has already been decided according a fixed ratio of the number of beds in the hospital. According to the same law, managers of hospitals in Japan should be physicians. Therefore, we assume that the other health care professionals at the hospital are allocated proportionally to the number of doctors and hospital size. The number of hospital beds cited from official statistics of the Japanese government [17] was used as an index of capital input.

As a production output, we obtained the number of discharged patients, weighted by a casemix-specific relative weight [13]. More specifically, the relative weight is a ratio of the average amount of resources required for the treatment of patients belonging to a casemix category, relative to that of all discharged cases. The weight reflects the relative severity of the disease, and subsequent resource use. Many Japanese hospitals offer outpatient as well as inpatient services, though we did not include the number of outpatients, mainly because of data limitation.

Finally, we used the hospital standardized mortality ratio (HSMR) to account for the quality of inpatient services. The HSMR is a ratio calculated by dividing the observed hospital mortality rate by the expected hospital mortality rate [18]. The prediction model that estimates the expected hospital mortality rate has an independent variable that takes the value 1 in the case of an in-hospital death, and zero otherwise. In the model, the number of in-hospital deaths of discharged cases was regressed using a logistic model of age, sex, disease category, use of surgical intervention, emergency status, co-morbidities at admission, and disease severity scores. Higher ratios of the HSMR mean that there was excess mortality for the patient’s conditions, suggesting poor quality of care.

### Regression of the estimated fixed effect in terms of hospital characteristics

The estimated values of hospital-specific fixed effects were further regressed using an ordinary least-squares linear model of the location and the specific function of each hospital in the community care system. Specifically, we chose five explanatory variables: the “advanced treatment hospital dummy variable", "casemix index", "number of physicians per 100,000 people living locally (i.e., physician density)”, “number of hospitals per 100,000 people living locally (i.e., hospital density)”, and “proportion of the local population aged 65 and over.” The status of an advanced treatment hospital was accredited only to main branch hospitals of university affiliation and national center hospitals for cancer and cardiovascular diseases. The status of advanced treatment hospital indicates that the hospital has a tertiary function with teaching and research duties. This prestigious status is expected to be associated with lower output given the same resource. A higher casemix index suggests that the hospital treated patients with more severe conditions, and should logically be associated with higher production. Local density of physicians and hospitals reflects the degree of competitiveness in the local market. High competitiveness should positively affect the production output, regardless of hospital efficiency. Finally, local demographics of population ageing would be a marker of greater local healthcare demand, which should be associated with larger production, regardless of hospital function and efficiency. These explanatory variables were recorded for the municipality unit in which the hospital was located.

Limdep 8.0 (Econometric Software, Inc.) and SPSS 19.0 (SPSS Japan, Inc.) software were used for all analyses.

## Results

### Estimation of efficiency

Descriptive statistics of the variables used in the estimation of technical efficiency are presented in Table 1, and the estimation results are presented in Table 2. The variables used in the Cobb–Douglas model (equation 1) were all statistically significant, and all sign directions were as expected. The inputs, the number of physicians and the number of beds, were both positive and statistically significant. The coefficient of the HSMR was significantly negative, suggesting that low mortality rates (or high-quality care) decreases output. The translog model (equation 2) yielded results similar to those obtained with the Cobb–Douglas model (equation 1). The 3-year average of the efficiency estimated with the Cobb–Douglas model (equation 1) was 0.586, while that estimated with the translog model (equation 2) was 0.610.

**Table 1:** Descriptive statistics used in the estimation of technical efficiency

	**Weighted number of inpatients**	**Number of physicians**	**Number of beds**	**HSMR**
Mean	2528.67	108	600	1.180
S.D.	1618.40	64	294	0.315
Maximum	7043.48	266	1475	2.243
Minimum	15.40	16	130	0.549

The data we used in the TFEM is a balanced panel dataset collected between 2005 and 2007 from 127 hospitals. The sample hospitals had an average of 600 beds, which is a relatively large number for Japanese hospitals.

**Table 2:** Results of technical efficiency estimation (N = 127 for 3 years)

	**Cobb–Douglas function**		**Translog function**	
	**(equation 1)**		**(equation 2)**	
	**Coefficient**	**Standard error**	**Coefficient**	**Standard error**
*ln *Number of physicians	0.384***	0.019	2.898***	0.304
*ln *Number of beds	0.665***	0.034	−0.525**	0.211
Hospital standardized mortality ratio	−0.208***	0.022	−0.172***	0.020
*ln *Number of physicians (squared)			0.438***	0.108
*ln *Number of beds (squared)			0.691***	0.115
*ln *Number of physicians x ln number of beds			−0.711***	0.118
Efficiency score	0.610		0.586	
Log likelihood	−66.852		−88.115	
Likelihood ratio test value	365.405***		322.880***	

***p < 0.01 **p < 0.05.

The results obtained with the TFEM show that all explanatory variables in the Cobb–Douglas function (equation 1) were statistically significant, and all sign directions were as expected. The inputs, the number of physicians and the number of beds, were both positive and statistically significant. The coefficient of the HSMR was significantly negative, suggesting that low mortality rates (or high quality of care) decreases output. The translog model (equation 2) yielded results similar to those obtained with the Cobb–Douglas model (equation 1).

### Estimation of hospital-specific fixed effects

**Figure 1:**
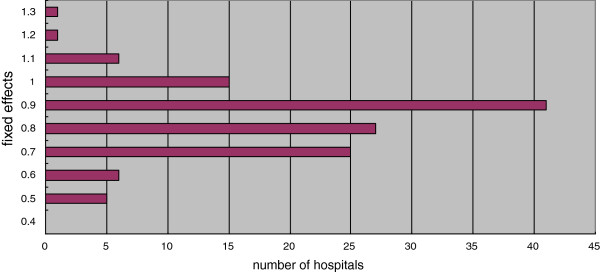
**Histogram of the estimated fixed-effect values.** The mean value of the dummy variable (α_*i*_) that complements the fixed effect was 0.784, and the standard deviation was 0.137. The minimum and maximum values were 0.437 and 1.212, respectively, and the maximum value was 2.77 times the minimum value. The peak of the distribution of values was around 0.9 as indicated in Figure 1.

**Figure 2:**
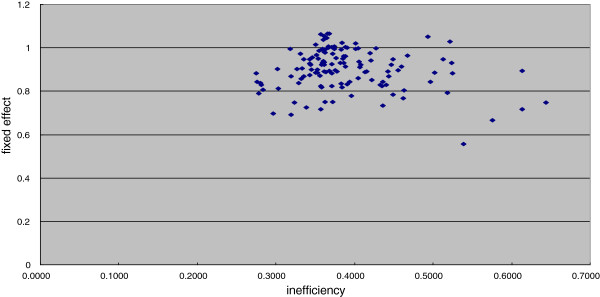
**Scatter plots of the averageinefficiency and fixed-effect values.** The scatter plot has a horizontal axis representing the average inefficiency score estimated by the TFEM (translog model) and a vertical axis indicating the fixed-effect estimator value. The two variables appear to be uncorrelated.

The mean value of the hospital-specific fixed effects (α_*i*_) was 0.784, the standard deviation was 0.137, and the range was between 0.437 and 1.212. The peak of the distribution of values was around 0.9 as depicted in Figure 1. Figure2is a plot of the time-invariant fixed-effect value versus the 3-year average of inefficiency for each hospital. The correlation between the estimated fixed-effect estimator and the efficiency score was −0.1631, and not statistically significant, suggesting statistical independence between the two estimates (Figure 2).

### Secondary regression analysis of the value of the fixed-effect variable

Descriptive statistics of explanatory variables are presented in Table 3, and results of the regression analysis are summarized in Table 4. The adjusted coefficient of determination (adjusted R^2^) was 0.15 and relatively low. Four out of six explanatory variables were statistically significant. With respect to the characteristics of the hospitals, the coefficient of the advanced treatment hospital dummy variable was negative, and statistically significant. The casemix index and hospital density had statistically significant positive coefficients. The remaining two explanatory variables, physician density and proportion of the local population aged 65 years or older, had positive coefficients of correlation as hypothesized, although they did not reach statistical significance. Model F statistics reached statistical significance, and the variance inflation factor of less than 10 indicates that there were no serious multi-collinearity problems.

**Table 3:** Descriptive statistics of explanatory variables used to predict hospital-specific fixed effects

	**Mean**	**S.D.**	**Min**	**Max**
Advanced treatment hospital	0.46	0.50	0.00	1.00
Casemix index	0.92	0.17	0.48	1.31
Number of doctors per unit of population	437.19	398.76	110.56	1891.03
Number of hospitals per unit of population	7.28	3.46	2.49	21.67
Proportion aged ≥ 65 years	0.19	0.03	0.11	0.31

The table gives descriptive statistics of the dataset used in ordinary least-squares regression. The sample size is 127 hospitals.

**Table 4:** Results of the ordinary least-squares regression analysis of the hospital-specific fixed effect

	**Coefficient**	**S.E.**	**Standardized coefficient**	**Variance inflation factor**
Constant term	0.332	0.109		
Advanced treatment hospital	−0.115	0.038	−0.416	2.877
Casemix index	0.455	0.107	0.554	2.505
Number of doctors per unit of population	0.004	0.004	0.103	1.453
Number of hospitals per unit of population	7.63E–05	0.000	0.221	1.211
Proportion aged ≥ 65 years	0.123	0.410	0.030	1.498
Adjusted R squared	0.147			
F-statistics	5.333			

***p < 0.01 **p < 0.05.

The results of ordinary least-squares regression were that three out of the five explanatory variables were statistically significant. In terms of the factors of hospital characteristics, status as an advanced treatment hospital and the Casemix index were significant. In terms of the hospital location, the number of hospitals per unit of population was statistically significant.

## Discussion

The 3-year average of the estimated efficiency was around 0.60, which was lower than 0.896 reported by Jacobs, Smith, and Street (2006), 0.78 by Kawaguchi (2008), and 0.79 by Takatsuka and Nishimura (2008) [8-10]. This discrepancy could be attributed to differences in the nature of sampled hospitals, the functional form of estimation models, and inclusion of a quality indicator in our model. Jacobs, Smith, and Street (2006) used a simple linear cost function with 4-year panel data of 185 samples from public hospitals in the United Kingdom [8]. Kawaguchi (2008) analyzed 5-year panel data of 862 municipal hospitals in Japan with a Cobb–Douglas cost function, accounting for patient characteristics and proxy indicators of care quality [9]. Takatsuka and Nishimura (2008) investigated the effects of introducing a new ordering system on efficiency with 5-year panel data of 408 municipal hospitals using a translog production function [10]. When we removed the quality indicator from our model, the 3-year average efficiency increased to 0.744, a level similar to that reported in the previously published studies described above. Thus, we speculate that previous studies may fail to discriminate the efficiency of quantity production from the quality of production. Additionally, Mutter *et al.* (2008) reported that the mortality rate of Medicare patients had a negative coefficient of correlation with the cost function, implying that higher quality production increased costs [19].

Previous economic studies of hospital production efficiency focused on estimated efficiency, and conducted a secondary regression analysis to identify factors related to the efficiency. Our idea to use the estimated fixed-effect value as an indicator of structural hospital properties of the production function is unique. The estimated hospital-specific fixed effects were statistically independent of estimated efficiency scores, and had wide variance across hospitals. The secondary regression analysis confirmed that the fixed-effect estimators were significantly associated with hospitals’ advanced functions and local competitiveness, as hypothesized. These results indicate that the numerical value of the fixed-effect estimator may reflect the unobserved time-invariant heterogeneity of hospitals that corresponds to the structural properties of their location and function.

Identifying the contribution of these structural properties to a hospital’s production function has an important policy implication, especially under rigid price regulation as is the case in Japan, Canada, and some European countries. Non-profit hospitals are expected to meet societal demands of high quality and quantity of service provision regardless of unprofitable external conditions [20]. Because inclusive payment mainly covers variable cost, non-profit hospitals with high functional and academic duties or those in remote rural areas often need complementary subsidization. However, subsidies discourage efficiency. Our new method may provide an approach for discriminating the contribution of external structural properties and efficiency per se, and for tailoring the finance method of non-profit hospitals under non-profitable external conditions without discouraging improvements in efficiency.

### Limitations and future directions

Similar to previous studies on hospital production efficiency, our study suffers several limitations. First, a relatively small sample size and a short time interval of 3 years may limit the generalizability and estimation efficiency of our results. Despite our best efforts to obtain the necessary information to construct our production function model, data in panel form were only available from 127 DPC hospitals. As a result, our sample is not representative of all Japanese hospitals, and reflects only advanced, acute-care hospitals. Greene (2005a) argued that when a fixed-effect model is used for data in a relatively short period, and a period of three years is short in this context, the estimated parameters may be biased and there may be an "incidental parameters" problem [6]. Chen, Schmidt, and Wang (2011) investigated the effects of the incidental parameter problem on the fixed-effect parameters [[21]]. The effect on E [*u*_*it*_/*ɛ*_*it*_] is still unclear. They also suggested that the incidental problem would be reduced by adopting data covering periods from 5 to 10 years [[21]]. Thus, our results and the usefulness of fixed-effect parameters in discriminating hospital structural properties should be confirmed with a larger and longer panel dataset.

Second, we used a simple production function as the first step in testing our hypothesis. Because a simple empirical model was used in this study, there is a possibility of the omitted variables problem, which may bias the estimation of time-variant component of hospital production efficiency. However, fixed effect model should allow us to obtain time-consistent component of productive efficiency more free from such misspecification, and we believe the second regression would tell how such time consistent characteristics of hospital production function were related to regional healthcare needs and demographic characteristics.

Finally, in our estimation, the minimum efficiency score was negative and the maximum efficiency score was not 1. The same problem was reported by Jacobs, Smith, and Street (2006) in their analysis of National Health Service hospitals in the United Kingdom using the same method [8]. This is apparently a limitation of statistical modeling, and further studies are needed to resolve this issue.

## Conclusions

Despite the limitations identified above, our analyses indicated that the estimated efficiency of Japanese acute-care hospitals was approximately 0.6, which is lower than the estimates made in previous studies that do not account for quality of services. We also examined the value of the fixed-effect constant terms as adjustments for unobserved heterogeneity, and confirmed a correlation between the fixed value and the function and location of the sample hospitals. The policy implications of these results are that a fixed-effect variable may be a promising tool in evaluating a hospital’s structural conditions, and in improving the fairness of reimbursement among hospitals under price regulation and inclusive-payment systems. We also hope that this concept can be applied to other types of reimbursement systems based on casemix classification. However, the proposed method and concept require further empirical investigation with a larger and longer hospital panel dataset.

## Competing interests

The authors declare that they have no competing interests.

## Authors’ contributions

HH conceived the study. HK conducted data analysis and interpretation. HK and HH co-wrote the paper. SM managed data collection. “All authors have read and approved the final manuscript.”

## Pre-publication history

The pre-publication history for this paper can be accessed here:

http://www.biomedcentral.com/1472-6963/12/334/prepub

## References

[B1] Schreyogg J Stargardt T Tiemann O Busse R Methods to determine reimbursement rates for diagnosis related groups (DRG): A comparison of nine European countries Health Care Manag Sci 2006 9 215 223 10.1007/s10729-006-9040-1 17016927

[B2] Hsiao WC Braun P Becker ER Managing Reimbursement in the 1990s 1990 New York: McGraw-Hill

[B3] w Center for Medicare and Medicaid Service: FY 2011 PPS Final Rule Home Page https://www.cms.gov/AcuteInpatientPPS/IPPS2011/list.asp#TopOfPage

[B4] Hollingsworth B The measurement of efficiency and productivity of health care delivery Health Econ 2008 17 Suppl 10 1107 1128 18702091 10.1002/hec.1391

[B5] Kumbhakar SC Lovell CA Stochastic Frontier Analysis 2000 Cambridge: Cambridge University Press

[B6] Greene W Fixed and random effects in stochastic frontier models J Prod Anal 2005 23 7 23 10.1007/s11123-004-8545-1

[B7] Greene W Reconsidering heterogeneity in panel data estimators of the stochastic frontier model J Econometrics 2005 126 269 303 10.1016/j.jeconom.2004.05.003

[B8] Jacobs R Smith PC Street A Measuring Efficiency in Health Care—Analytic Techniques and Health Policy 2006 Cambridge: Cambridge University Press

[B9] Kawaguchi H Measurement of Efficiency in Health Care—Its Methods and Issues 2008 Tokyo: Keiso Shobo

[B10] Takatsuka N Nishimura S Investigation of the effect of the ordering system on the hospital productivity and efficiency Jpn J Health Econ Pol 2008 20 15 33

[B11] Newhouse JP Frontier estimation: how useful a tool for health economics? J Health Econ 1994 13 Suppl 3 317 322 10138857 10.1016/0167-6296(94)90030-2

[B12] Kawaguchi H Hashimoto H Matsuda S Study about evaluation of hospital efficiency and function used by DPC data set. [in Japanese] The Journal of Health Care and Society [Iryo to Shakai] 2010 20 Suppl 1 23 34

[B13] Matsuda S Diagnosis procedure combination: The Japanese approach to casemix The Globalization of Managerial Innovation in Health Care 2008 Cambridge: Cambridge University Press

[B14] Campbell JC Ikegami N The Art of Balance in Health Policy—Maintaining Japan's Low-Cost, Egalitarian System 1998 Cambridge: Cambridge University Press

[B15] Jondrow J Lovell CAK Materov IS Schmidt P On the estimation of technical inefficiency in the stochastic frontier production function model J Econ 1982 19 233 238

[B16] Yodosha Directory editing group Directory of teaching hospitals 2005 Tokyo: Yodosha

[B17] Ministry of Health, Labour and Welfare Hospital Report 2005 Tokyo: Health and Welfare Statistics Association

[B18] Miyata H Hashimoto H Horiguchi H Matsuda S Motomura N Takamoto S Performance of in-hospital mortality prediction models for acute hospitalization: hospital standardized mortality ratio in Japan BMC Health Serv Res 2008 8 229 10.1186/1472-6963-8-229 18990251 PMC2606685

[B19] Mutter RL Rosko MD Wong HS Measuring hospital inefficiency: The effects of controlling for quality and patient burden of illness Health Services Res 2008 43 Suppl 6 1992 2013 10.1111/j.1475-6773.2008.00892.x PMC2614001 18783458

[B20] Zweifel P Breyer F Health Economics 1997 Oxford: Oxford University Press

[B21] Chen Y-Y Schmidt P Wang H-J Consistent Estimation of the Fixed Effects Stochastic Frontier Modelhttp://economics.uwo.ca/workshop/applied/schmidt_nov11.pdf

